# Calcitriol Treatment Decreases Cell Migration, Viability and β-Catenin Signaling in Oral Dysplasia

**DOI:** 10.3390/cimb46040191

**Published:** 2024-04-02

**Authors:** Daniel Peña-Oyarzún, Constanza Guzmán, Catalina Kretschmar, Vicente A. Torres, Andrea Maturana-Ramirez, Juan Aitken, Montserrat Reyes

**Affiliations:** 1 Faculty of Odontology and Rehabilitation Sciences, Universidad San Sebastián, Los Leones Campus, Santiago 7510157, Chile; 2 Department of Pathology and Oral Medicine, Faculty of Odontology, Universidad de Chile, Santiago 8380544, Chile; 3 Institute for Research in Dental Sciences, Faculty of Odontology, Universidad de Chile, Santiago 8380544, Chile; 4 Millennium Institute on Immunology and Immunotherapy, Universidad de Chile, Santiago 8380453, Chile; 5 Advanced Center for Chronic Diseases (ACCDiS), Universidad de Chile, Santiago 8380494, Chile

**Keywords:** calcitriol, oral cancer, oral dysplasia, β-catenin

## Abstract

Nearly 90% of oral cancers are characterized as oral squamous cell carcinoma (OSCC), representing the sixth most common type of cancer. OSCC usually evolves from oral potentially malignant disorders that, in some cases, are histologically consistent with a oral dysplasia. The levels of 1α,25 dihydroxyvitamin D3 (1,25-(OH)2D3; calcitriol), the active form of vitamin D3, have been shown to be decreased in patients with oral dysplasia and OSCC. Moreover, treatment with 1,25-(OH)2D3 has been proven beneficial in OSCC by inhibiting the Wnt/β-catenin pathway, a signaling route that promotes cell migration, proliferation, and viability. However, whether this inhibition mechanism occurs in oral dysplasia is unknown. To approach this question, we used dysplastic oral keratinocyte cultures and oral explants (ex vivo model of oral dysplasia) treated with 1,25-(OH)2D3 for 48 h. Following treatment with 1,25-(OH)2D3, both in vitro and ex vivo models of oral dysplasia showed decreased levels of nuclear β-catenin by immunofluorescence (IF) and immunohistochemistry (IHC). Consistently, reduced protein and mRNA levels of the Wnt/β-catenin target gene survivin were observed after treatment with 1,25-(OH)2D3. Moreover, 1,25-(OH)2D3 promoted membranous localization of E-cadherin and nuclear localization of vitamin D receptor (VDR). Functionally, DOK cells treated with 1,25-(OH)2D3 displayed diminished cell migration and viability in vitro.

## 1. Introduction

Oral cancer is a malignant neoplasm that arises as a lesion of primary origin in the oral tissues that line the oral cavity [1,2]. The incidence of oral cancer has increased dramatically worldwide in recent years, and only 40–50% of patients survive at 5 years, leading to the fact that oral cancer is a major problem of global public health [3]. About 90% of oral cancers originate in the stratified, non-keratinized epithelium of the oral mucosa, which is the reason for its denomination as oral squamous cell carcinoma (OSCC), and the main risk factors accounting for it include consumption of tobacco and alcohol [3,4]. OSCC is usually preceded by oral potentially malignant disorders (OPMDs), a group of mucosal abnormalities with increased risk of developing oral cancer, such as leukoplakia, erythroplakia, proliferative verrucous leukoplakia, and oral lichen planus, among others [5]. Oral epithelial dysplasia is a histological grade of some OPMDs characterized by loss of cell polarity, sharp lateral margins, and abnormally large hyperchromatic nuclei, as well as other apoptotic and architectural features [6]. Hence, early detection of oral dysplasia can minimize OSCC morbidity, while avoiding the detrimental side effects associated with OSCC treatment [4,7].

We and others have previously shown that the Wnt/β-catenin pathway, also known as the Wnt canonical pathway, is upregulated in oral dysplasia biopsies and dysplastic oral keratinocytes [8,9,10,11]. In normal oral epithelial cells, β-catenin is continuously targeted for proteasomal degradation by a protein destruction complex formed by casein kinase 1α (CK1α), glycogen synthase kinase 3β (GSK-3β), tumor suppressor protein adenomatous polyposis coli (APC) and Axin, resulting in a switched-off Wnt/β-catenin pathway [12,13] β-catenin is targeted for degradation by phosphorylation at Ser33/Ser37/Thr41 [12,13]. Remarkably, in oral dysplastic cells, the Wnt/β-catenin pathway is upregulated, a phenomenon associated with increased Wnt ligand secretion, which ultimately blocks β-catenin phosphorylation and its subsequent degradation [9,14]. Cytoplasmic stabilization of this non-phosphorylated form of β-Catenin (also known as transcriptionally active) is followed by its translocation to the nucleus, allowing the transcription of TCF/LEF target genes, which are associated with cell proliferation and cell viability, such as survivin and cyclin D1 [9]. Indeed, we have demonstrated in both in vitro and ex vivo models of oral dysplasia that massive nuclear localization of β-catenin occurs in a Wnt3a-dependent manner [9,14]. These observations support the notion that aberrant activation of the canonical Wnt signaling pathway is a recurring event in oral tumorigenesis.

Recent studies indicate that vitamin D3 and its most active metabolite, 1α,25 dihydroxyvitamin D3 (also known as calcitriol or “1,25-(OH)2D3”), have multiple therapeutic anti-inflammatory, antioxidant, and anti-carcinogenic effects [15,16,17]. These actions are mediated by the vitamin D receptor (VDR), a transcriptional factor activated by intracellular binding of 1,25-(OH)2D3 [18,19,20]. The use of 1,25-(OH)2D3 as a novel adjuvant for cancer treatment has been demonstrated in several neoplasms, including colorectal and breast cancer, among others [15,16,21,22]. Epidemiologic studies have suggested that low vitamin D status (<30 ng/mL) is associated with an increased risk of cancer and poorer prognosis [17,23]. Interestingly, lower serum levels of 1,25-(OH)2D3 have been associated with a higher incidence of oral dysplasia and OSCC [24,25].

The mutual regulation between 1,25-(OH)2D3 and β-catenin signaling has been proposed. 1,25(OH)2D3 inhibits Wnt/β-catenin signaling by several mechanisms at different points along the pathway. Previous work has demonstrated mainly that 1,25(OH)2D3 inhibits β-catenin transcriptional activity by promoting VDR binding to β-catenin, promoting the translocation of β-catenin from the nucleus to the plasma membrane, and inducing of E-cadherin expression in several cancers [15,22,26]. Intriguingly, whether 1,25-(OH)2D3 disrupts the Wnt/β-catenin pathway in oral dysplasia, is unknown. Therefore, this study sought to investigate the effects of 1,25-(OH)2D3 on cell migration, cell viability, and β-catenin-dependent signaling in oral dysplastic models in vitro and ex vivo.

## 2. Materials and Methods

### 2.1. Materials

Mouse monoclonal anti-β-catenin (M3539) was from DAKO Agilent (Santa Clara, CA, USA), and rabbit monoclonal anti-non-phosphorylated β-catenin (lacking phosphorylation on residues Ser33/37/Thr41; 88145) was from Cell Signaling Technology (Denver, MA, USA). E-cadherin and VDR mouse monoclonal antibodies were from Santa Cruz Biotechnology (Dallas, TX, USA). Ki-67 (SP6) rabbit monoclonal was from Cell Marque (Rocklin, CA, USA). Alexa Fluor 488 and Alexa Fluor 568 conjugated secondary antibodies were from Invitrogen (Carlsbad, CA, USA). 1α,25-Dihydroxyvitamin D3 and Lithium Chloride (SC203110A) were from Santa Cruz Biotechnology (Dallas, TX, USA). Goat anti-rabbit and goat anti-mouse antibodies coupled to horseradish peroxidase (HRP) were from Bio-Rad Laboratories (Hercules, CA, USA). Tissue culture medium, antibiotics, and fetal bovine serum (FBS) were from Corning Mediatech (Manassas, VA, USA). The EZ-ECL chemiluminescent substrate was from Pierce Chemical (Rockford, IL, USA). Tissue culture medium, antibiotics, and fetal bovine serum (FBS) were from Corning Mediatech (Manassas, VA, USA).

### 2.2. Cell Culture

The dysplastic oral keratinocytes (DOK) cell line was obtained from Sigma-Aldrich (ECACC #94122104; St. Louis, MO, USA). DOK cells derive from a moderate dysplasia at the tongue of a 57-year-old man with oral squamous cell carcinoma. Cells were cultured in DMEM-high glucose, supplemented with penicillin (10,000 U/mL) and streptomycin (10 μg/mL). For treatment, DOK cells were stimulated with 0.1 µM 1,25-(OH)2D3 or vehicle (isopropanol) for 48 h in culture media.

### 2.3. Immunofluorescence

Cells were grown on glass coverslips and fixed with 4% formaldehyde in PBS for 15 min. Permeabilization was done with 0.1% Triton X-100 in PBS for 15 min. After washing with PBS, samples were blocked with 5% bovine serum albumin (BSA) in PBS for 1 h and incubated with primary antibodies (diluted in 5% BSA) overnight at 4 °C. Following a PBS wash, samples were incubated with secondary antibodies for 1 h at room temperature, and stained with Hoechst 33342 (diluted 1:10,000 in PBS) for 10 min. Finally, samples were mounted using the DAKO mounting medium and visualized by fluorescence microscopy, using a Nikon C2 Plus confocal microscope.

### 2.4. Oral Explant

Tissue biopsies were obtained from three donor patients diagnosed with oral dysplasia who were attending the Dental Clinic, Faculty of Dentistry, University of Chile, Santiago, Chile. Ethical human agreement was mandatory, as requested by the Comité Ético Científico, Faculty of Dentistry, Universidad de Chile. All subjects were evaluated for the presence of clinical oral lesions, such as leukoplakia, erythroplakia, and erythroleukoplakia, which were histologically diagnosed as dysplasia.

Oral tissues were divided into two pieces and cultured in DMEM medium with either 0.1 µM 1,25-(OH)2D3 or vehicle for 48 h. Then, samples were prepared for histology and immunohistochemistry.

### 2.5. Measurement of Serum 25(OH)D

Serum 25-hydroxyvitamin D3 (the precursor of 1,25-(OH)2D3, which is also known as calcidiol or “25(OH)D”) was measured in donor patients by electrochemiluminescence immunoassay (ECLIA, Roche, Basel, Switzerland) for total vitamin D (25-OHD, D2, and D3). Vitamin D deficiency was defined as <30 ng/mL 25(OH)D.

### 2.6. Immunohistochemistry

Oral explants were fixed in 10% formalin and included in paraffin blocks. Sections of the included samples (3 µm) were deparaffinized with xylene and rehydrated with ethanol (decreasing concentrations) and distilled water. Antigen retrieval was done with sodium citrate buffer (pH 6.0), while endogenous peroxidase activity was inactivated with 3% hydrogen peroxide in methanol for 10 min. Sections were blocked with horse serum for 30 min and incubated with primary antibodies against β-catenin, E-cadherin, VDR, and Ki-67 (overnight at 4 °C). Sections were incubated with biotinylated secondary antibodies for 30 min at 37 °C and then with peroxidase-conjugated streptavidin (Universal Detection System Vectastain Elite Kit wide spectrum ABC-HRP, RTU, Vector-USA, EE. UU) for 30 min at 37 °C. The reaction was finally visualized with diaminobenzidine (DAB) and stained with Harris hematoxylin (HE).

### 2.7. SDS-PAGE and Western Blot

Cells were washed with ice-cold PBS and homogenized in Tissue Protein Extraction Reagent (T-PER; Thermo Fisher Scientific, Waltham, MA, USA) lysis buffer supplemented with protease and phosphatase inhibitors. Total protein extracts were resolved by denaturing polyacrylamide gel electrophoresis (SDS-PAGE) and transferred to nitrocellulose membranes for Western blotting using a Trans-Blot Turbo System (Bio Rad, Hercules, CA, USA). Membranes were blocked with 5% non-fat milk in 0.1% Tween-TBS for 1 h and then incubated with primary antibodies overnight. Primary antibodies were detected with HRP-conjugated antibodies using a chemiluminescence EZ-ECL system as the HRP enzyme substrate.

### 2.8. Viability Analysis

DOK cells were seeded in 96-well plates at a density of 10,000 cells per well. After 24 h, a 20:1 mixture of MTS: PMS (MTS^®^ kit; Promega, Madison, WI, USA) was added to each well and incubated for 1 h at 37 °C. The reaction was stopped with 10% SDS. Finally, the reduction of the MTS compound in formazan was determined by measuring the absorbance at 490 nm.

### 2.9. Migration Assays

Transwell migration assays were performed in Boyden Chambers (Transwell Costar, 6.5 mm diameter and 8 μm pore diameter; Sigma-Aldrich, St. Louis, MO, USA). The outer layers of the inserts were pre-coated with 2 μg/mL fibronectin. 50,000 cells per condition were re-suspended in serum-free DMEM medium and plated into the top chamber. Complete medium with 10% FBS was added to the bottom chamber. After 5 h, inserts were washed with distilled water and simultaneously stained and fixed with 0.1% Crystal Violet in methanol.

### 2.10. RNA Isolation and RT-qPCR

RNA was extracted with an RNeasy Kit ™ (Qiagen, Hilden, Germany). Reverse transcription of 1 μg RNA per sample was performed using the High Capacity cDNA Reverse Transcription Kit (Applied Biosystems, Waltham, MA, USA). Quantitative amplification of ciclin-D1 (primers 5′-CCACCTGTCCCACTCCTACGAT-3′; 5′-GCAGGGCCGTTGGGTAGAAA-3′), survivin (primers 5′-GCTTCGCTGGAAACCTCTGGA-3′; 5′-TCTGGGCAGATGGCTGTTGG-3′), GAPDH (primers 5′-ACCCACTCCTCCACCTTTGA-3′;5′-CTGTTGCTGTAGCCAAATTCGT-3′) cDNAs was done with Fast SYBR^®^ Green Master Mix (Applied Biosystems, Waltham, MA, USA) and analyzed with a StepOne Real-Time PCR system (Applied Biosystems, Waltham, MA, USA).

### 2.11. Statistical Analysis

Given that exploratory analysis showed a normal distribution, *t*-test, and ANOVA were used. Values are graphically depicted as the mean ± SEM (standard deviation of the mean; from at least three independent experiments). A *p* < 0.05 was considered statistically significant. GraphPad Prism software version 6 was used for statistical analysis.

## 3. Results

### 3.1. Effects of 1,25-(OH)2D3 on β-Catenin Localization and the Expression of Target Genes in Dysplastic Oral Keratinocytes

**Figure 1:**
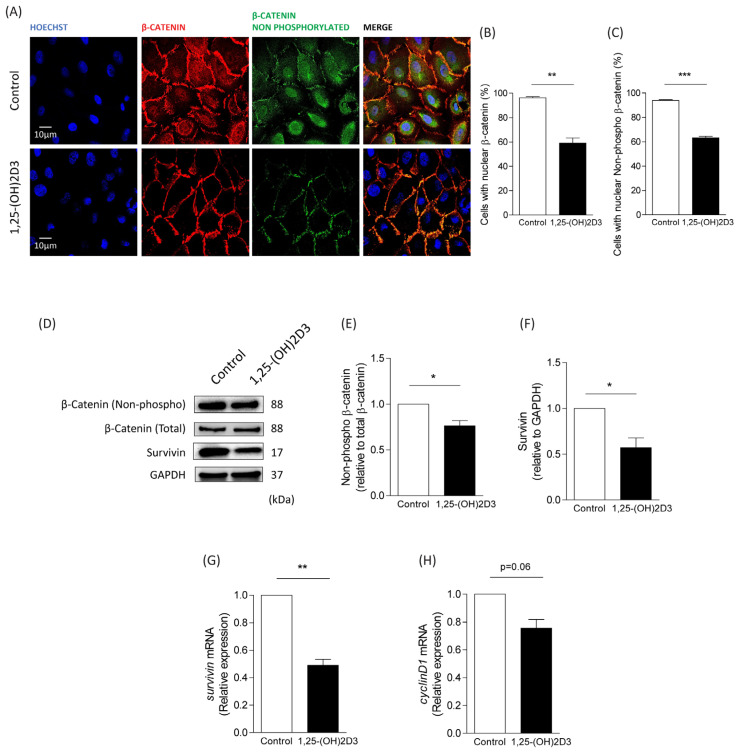
1,25-(OH)2D3 treatment decreases nuclear localization of β-catenin and expression of target genes in dysplastic oral cells. DOK cells were incubated with 1,25-(OH)2D3 or isopropanol (as vehicle control) for 48 h and used for subsequent analysis. (**A**–**C**) Subcellular localization of total β-catenin and non-phosphorylated (Ser33/Ser37/Thr41, transcriptionally active) β-catenin in DOK cells exposed to 0.1 µM of 1,25-(OH)2D3. Representative confocal microscope images from three independent experiments are shown in (**A**), using Hoechst for nuclear staining, while the percentage of cells with nuclear localization of total β-catenin and non-phosphorylated (Ser33/Ser37/Thr41, transcriptionally active) β-catenin is graphically depicted in (**B**,**C**), respectively (mean ± SEM; *t*-test; *** *p* ≤ 0.001; ** *p* ≤ 0.01; *n* = 3). (**D**–**F**) Western blot analysis of DOK cells treated with 1,25-(OH)2D3. Survivin, total and non-phosphorylated β-catenin, and GAPDH were blotted with specific antibodies. Representative blot images from 3 independent experiments are shown in (**D**), while relative levels of non-phosphorylated β-catenin (relative to total β-catenin) and survivin (relative to GAPDH) were quantified by scanning densitometry and graphically presented in (**E**,**F**), respectively (mean ± SEM; *t*-test; * *p* ≤ 0.05; *n* = 3). (**G**,**H**) Relative survivin and cyclin D1 mRNA levels were quantified by RT-qPCR (mean ± SEM; *t*-test; ** *p* ≤ 0.01; *n* = 3).

Following 48 h treatment with 0.1 µM 1,25-(OH)2D3, using isopropanol as a vehicle control, DOK cells were analyzed by immunofluorescence for β-catenin localization. In doing so, we found that 58% of cells showed nuclear β-catenin staining, as compared with the 96% of control vehicle-treated cells (Figure 1A,B). Accordingly, 61% of DOK cells treated with 1,25-(OH)2D3 presented nuclear localization of non-phosphorylated (Ser33/Ser37/Thr41, transcriptionally active) β-catenin, while 94% of cells treated with vehicle showed nuclear localization of non-phosphorylated β-catenin (Figure 1A,C). This suggests that 1,25-(OH)2D3 prevents nuclear translocation of both total and transcriptionally active β-catenin in an in vitro model of oral dysplasia. To confirm these observations, total protein levels of both β-catenin and its target gene, survivin, were assessed by Western blotting of vehicle- and 1,25-(OH)2D3-treated DOK cells. Treatment with 1,25-(OH)2D3 decreased protein levels of both non-phosphorylated β-catenin (relative to total β-catenin; Figure 1D,E) and survivin (relative to GAPDH; Figure 1D,F). Consistently, DOK cells treated with 1,25-(OH)2D3 displayed lower mRNA expression of survivin (*p* < 0.001; Figure 1G) and cyclin D1 (*p* = 0.06; Figure 1H). Taken together, these results suggest that treatment of dysplastic oral cells with 1,25-(OH)2D3 leads to decreased β-catenin activity.

### 3.2. Effects of 1,25-(OH)2D3 on E-Cadherin and VDR Localization in Dysplastic Oral Keratinocytes

**Figure 2:**
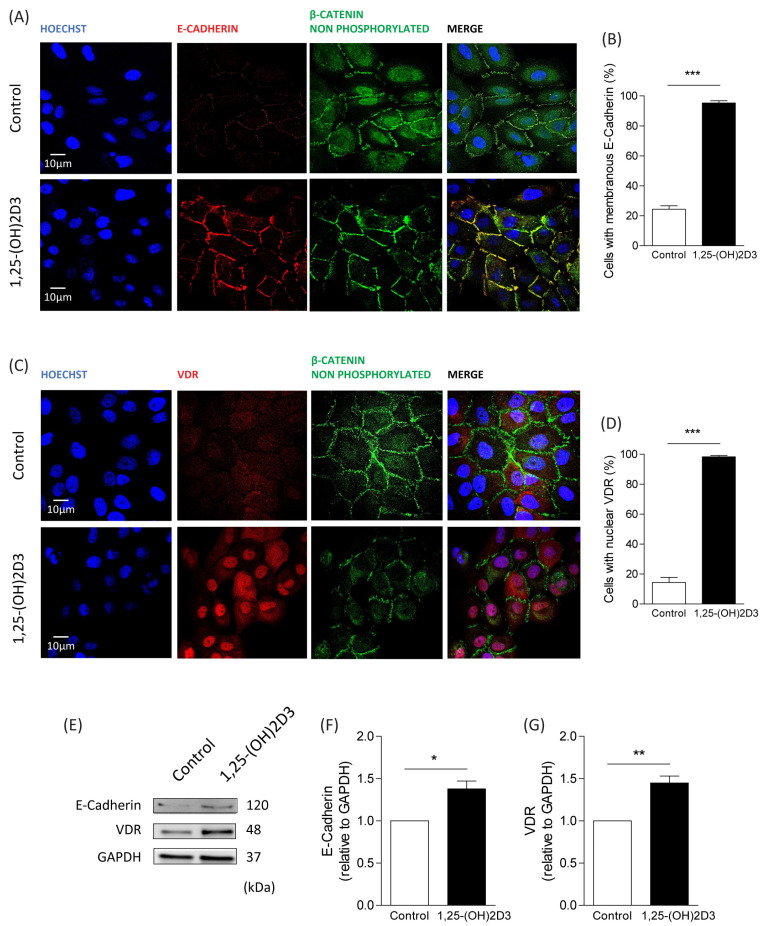
Increased expression of E-cadherin and VDR in oral dysplastic keratinocytes treated with 1,25-(OH)2D3. DOK cells were incubated with 1,25-(OH)2D3 (isopropanol as vehicle control) for 48 h. (**A**–**D**) Subcellular localization of E-cadherin, VDR, and non-phosphorylated (Ser33/Ser37/Thr41, transcriptionally active) β-catenin in DOK cells exposed to 0.1 µM of 1,25-(OH)2D3. Representative confocal microscope images, using Hoechst for nuclear staining, are shown in (**A**,**C**), while the percentage of cells with membranous localization of E-cadherin and nuclear VDR are graphically depicted in (**B**,**D**), respectively. Quantifications were obtained as described in the materials and methods, using the ImageJ software version 2.15.1, and data are shown as the average from three independent experiments (mean ± SEM; *t*-test; *** *p* ≤ 0.001; *n* = 3). (**E**–**G**) Western blot analysis of DOK cells treated with 1,25-(OH)2D3. E-cadherin, VDR, and GAPDH were blotted with specific antibodies. Representative images from 3 independent experiments are shown in (**E**), while densitometry analysis of E-cadherin and VDR levels (relative to GAPDH) is graphically depicted in (**F**,**G**), respectively (mean ± SEM; *t*-test; * *p* ≤ 0.05; ** *p* ≤ 0.01; *n* = 3).

We examined the membranous localization of E-cadherin in 1,25-(OH)2D3-treated and vehicle-treated DOK cells by immunofluorescence. We found that following treatment with 1,25-(OH)2D3, 95% of cells displayed membrane-localization of E-cadherin, which differed from the 24% observed in vehicle-treated cells (Figure 2A,B). This indicates that 1,25-(OH)2D3 increases E-cadherin expression at the cell membrane in oral dysplastic cells.

We then explored whether 1,25-(OH)2D3 promotes nuclear translocation of the intracellular receptor VDR. Compared to vehicle-treated DOK cells, which displayed 14% of cells with nuclear VDR, treatment of DOK cells with 1,25-(OH)2D3 showed 98% of cells with nuclear VDR (Figure 2C,D). This suggests that 1,25-(OH)2D3 increases nuclear translocation of VDR in oral dysplastic cells. Importantly, increased membranous localization of E-cadherin and nuclear translocation of VDR were found to be correlated with decreased nuclear β-catenin (Figure 2A,C).

Finally, we determined whether 1,25-(OH)2D3 also modulates E-cadherin and VDR total protein levels. To achieve this, DOK cells were stimulated with either 1,25-(OH)2D3 or control vehicle, and samples were analyzed by Western blotting. We observed that 1,25-(OH)2D3 led to increased protein levels of both E-cadherin and VDR (relative to GAPDH; Figure 2E–G). This indicates that 1,25-(OH)2D3 not only regulates E-cadherin and VDR localization in oral dysplastic cells but also enhances their protein levels.

### 3.3. Effects of 1,25-(OH)2D3 on Migratory Capacity and Viability in Dysplastic Oral Keratinocytes

**Figure 3:**
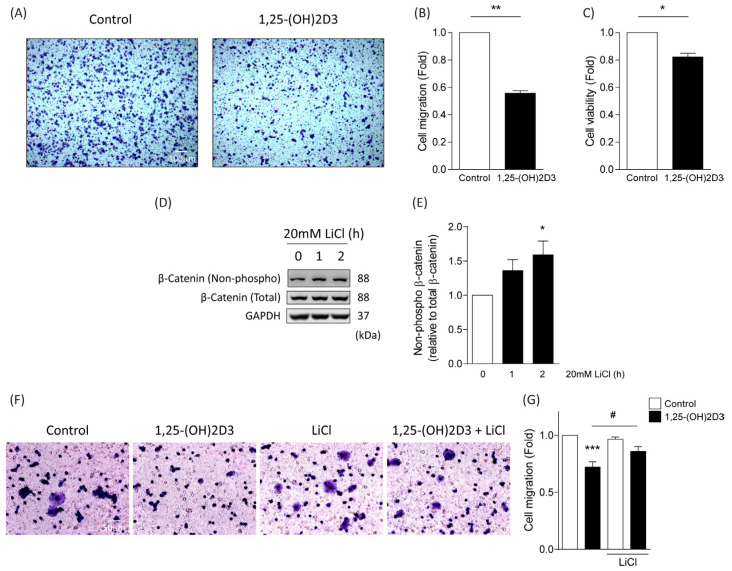
1,25-(OH)2D3 decreases cell migration and viability in oral dysplastic keratinocytes. (**A**,**B**) DOK cells were allowed to migrate for 300 min in Transwell chambers in the presence of either 0.1 µM 1,25-(OH)2D3 or vehicle. Cells that migrated were stained with crystal violet. Representative images are shown in (**A**), and graph that represents the averages of three independent experiments is depicted in (**B**) (mean ± SEM; *t*-test; ** *p* ≤ 0.01; *n* = 3). (**C**) Cell viability of DOK treated with either 0.1 µM 1,25-(OH)2D3 or vehicle was evaluated with the MTS^®^ kit, and formazan formation was measured at 490 nm (mean ± SEM; *t*-test; * *p* < 0.05; *n* = 3). (**D**,**E**) Western blot analysis of DOK cells treated with 20 mM lithium chloride (LiCl) for 1 or 2 h. Non-phosphorylated β-catenin, total β-catenin, and GAPDH were blotted with specific antibodies. Representative images from 3 independent experiments are shown in (**D**), and relative levels of non-phosphorylated β-catenin (relative to total β-catenin) are depicted in (**E**). (mean ± SEM; ANOVA; * *p* ≤ 0.05; *n* = 3). (**F**,**G**) Transwell cell migration assay was performed in DOK cells in the presence of either 0.1 µM 1,25-(OH)2D3 or vehicle control (48 h), and treated with 20 mM LiCl for 2 h. Representative images of cells stained with crystal violet are shown in (**F**), and quantification of migration fold from three independent experiments is graphically presented in (**G**) (mean ± SEM; ANOVA; *** *p* ≤ 0.001 vs. control, # *p* ≤ 0.05 vs. 1,25-(OH)2D3; *n* = 3).

Since 1,25-(OH)2D3 increased membranous localization of E-cadherin in DOK cells (Figure 2A,B), suggesting a more stable cell-to-cell adhesion phenotype, we set out to determine whether cell migration might be affected by 1,25-(OH)2D3. Indeed, treatment of DOK cells with 1,25-(OH)2D3 showed a 50% decrease in cell migration in a Transwell assay, compared with DOK cells treated with vehicle (Figure 3A,B).

On the other hand, given that 1,25-(OH)2D3 reduced protein and mRNA levels of survivin (Figure 1E–G), which is a critical protein involved in cell viability and apoptosis, we analyzed whether 1,25-(OH)2D3 impacted cell viability. DOK cells treated with 1,25-(OH)2D3 presented a 20% reduction in cell viability in an MTS assay, compared with vehicle-treated cells (Figure 3C). To assess whether these effects were due to reduced β-catenin activity induced by 1,25-(OH)2D3, we used lithium chloride (LiCl), which is known to inhibit β-catenin degradation. Activation of β-catenin in DOK cells was observed 2 h after treatment with 20 mM LiCl (Figure 3D,E). The use of LiCl prevented the decrease in migration observed following 1,25-(OH)2D3 treatment, suggesting that activation of β-catenin is sufficient to restore migration of cells challenged with 1,25-(OH)2D3 (Figure 3F,G). Collectively, these results suggest that 1,25-(OH)2D3 reduces cell migration and viability of oral dysplastic cells, and that β-catenin activation prevents the effects of 1,25-(OH)2D3 on cell migration.

### 3.4. Effects of 1,25-(OH)2D3 on Oral Explant from Tissues of Donor Patients with Oral Dysplasia

**Figure 4:**
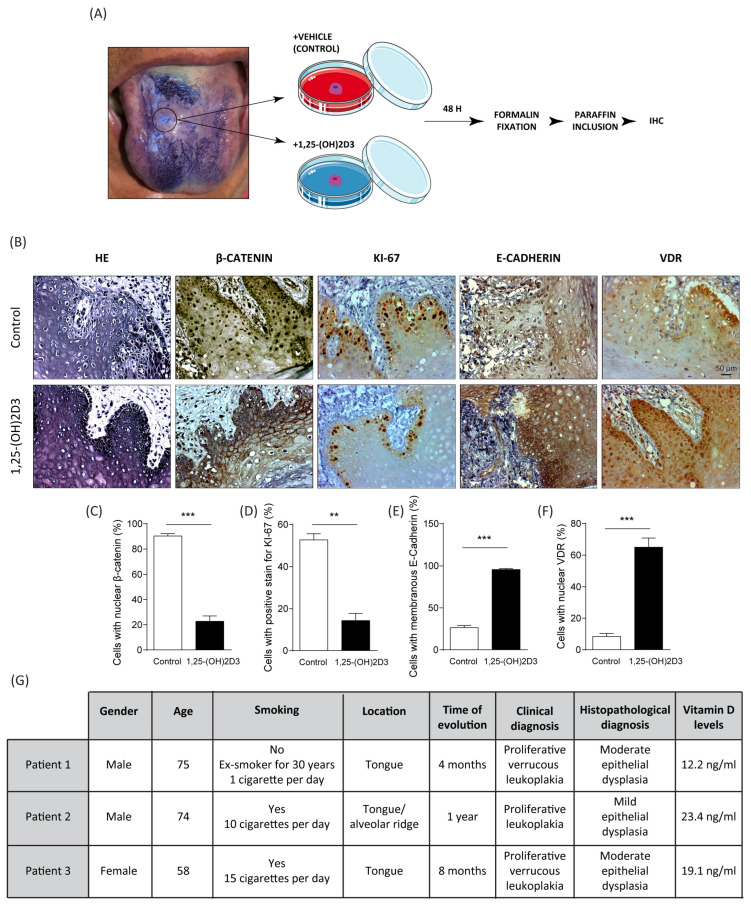
1,25-(OH)2D3 decreases nuclear β-catenin and Ki-67 and increases E-cadherin and VDR expression in oral dysplasia tissues. (**A**) Oral tissues from three patients with dysplasia were divided into two pieces and cultured in DMEM medium with either 0.1 µM 1,25-(OH)2D3 or isopropanol (vehicle) for 48 h. Oral explants were fixed in 10% formalin and included in paraffin blocks. (**B**–**F**) HE and immunohistochemistry of β-catenin, Ki-67, E-cadherin, and VDR in oral dysplasia. Representative images were photographed at 40× using an Olympus CX41 microscope and visualized with the Micrometrics SE Premium software version 4.5.1 (**C**), while quantification of the percentage of cells with nuclear β-catenin (**D**), positive KI-67 staining (**E**), membranous E-Cadherin (**F**), and nuclear VDR (**G**) were analyzed with the ImageJ software version 2.15.1 (** *p* ≤ 0.01 and *** *p* ≤ 0.001 vs. control; *n* = 3). (**G**) Clinical-demographic characteristics of the patients who voluntarily participated in this study.

To corroborate our results, we generated oral explant cultures using tissues from OPMD patients with histological diagnosis of oral dysplasia. Tissue samples obtained from patients were kept in DMEM medium until their arrival at the laboratory. Each sample was cut into two halves: one half was treated with 1,25-(OH)2D3 (0.1 μM) for 48 h, while the other half was incubated with vehicle alone. Samples were then fixed in 10%-buffered formalin for 24 h, before being embedded in paraffin (Figure 4A). 3 μm sections were made, and the subcellular localization of β-catenin, Ki-67 (a cell proliferation marker), E-cadherin, and VDR was determined by immunohistochemistry (Figure 4B).

Compared to explants treated with vehicle, explants treated with 1,25-(OH)2D3 showed reduced nuclear localization of β-catenin (Figure 4C) and expression of Ki-67 (Figure 4D), as well as increased membranous expression of E-cadherin (Figure 4E) and nuclear VDR (Figure 4F). These ex vivo results are consistent with those observed in vitro and collectively suggest that 1,25-(OH)2D3 reduces β-catenin activation, decreases cell proliferation, increases VDR nuclear translocation, and increases cell-to-cell adhesion. The clinical-demographic characteristics of the patients who voluntarily participated in this study are presented in Figure 4G. Interestingly, all three patients had low serum 25(OH)D levels (<30 ng/mL), suggesting that vitamin D deficiency may be associated to oral dysplasia in these patients.

## 4. Discussion

We and others previously showed that aberrant activation of the Wnt/β-catenin pathway is a recurrent phenomenon in oral dysplasia [8,11,27]. However, no information is available about factors that might negatively target this phenomenon, with the possibility of therapeutic applications. In this regard, it becomes relevant to consider the feasibility of 1,25-(OH)2D3 (calcitriol), which has been widely reported to target this pathway in different cancer models [17,18,19,20,22,28], although its effects have not been explored in OPMDs with histological diagnosis of oral dysplasia. Unlike other vitamins, such as vitamin A and C, vitamin D has shown remarkable antineoplastic effects in in vitro and in vivo oral cancer models [29]. Indeed, a systematic review that analyzed 80 studies published between 1959 and 2022 [29] showed improvement in the number and severity of oral lichen planus lesions in patients prescribed with vitamin D supplements [30], reduced tumor volume of oral cancer xenografts in mice co-treated with erlotinib (an epidermal growth factor receptor inhibitor) and 1,25-(OH)2D3 [16], and synergic effects of 5-fluorouracil and 1,25(OH)2D3 on human oral squamous cell carcinoma lines (C152) viability [31]. Moreover, other preclinical studies have shown that 1,25-(OH)2D3 can inhibit OSCC growth in vivo [16,21,25]. The purpose of this study was to evaluate the effects of 1,25-(OH)2D3 on cell migration, cell viability, and β-catenin activity in oral dysplasia. We revealed that 1,25(OH)2D3 reduced nuclear localization of both total and transcriptionally active β-catenin, which was in line with reduced expression of β-catenin target genes, including survivin and cyclin D1, at both mRNA and protein levels.

Several reports have demonstrated the inhibitory action of 1,25-(OH)2D3 on the Wnt/β-catenin pathway in different types of tumors, evidenced by the decreased formation of transcriptional TCF-β-catenin complexes and increased expression of Wnt antagonist [26,32]. In this work, we demonstrated that 1,25-(OH)2D3 increased the expression of the tumor suppressor protein E-cadherin, while decreasing the nuclear expression of non-phosphorylated β-catenin, in in vitro and ex vivo models of oral dysplasia. In breast and colon cancer, it has been shown that 1,25-(OH)2D3 increases the expression of E-cadherin, inducing translocation of β-catenin from the nucleus to the membrane, thus decreasing its transcriptional activity [22,26,33]. On the other hand, we found a decrease in the proliferation marker Ki-67 in explants treated with 1,25-(OH)2D3 compared with vehicle. Similar results have been found in skin keratinocytes, where treatment with 1,25(OH)2D3 inhibits β-catenin activity, thereby decreasing cell proliferation [19]. We also demonstrated that 1,25-(OH)2D3 induces expression of the nuclear receptor of vitamin D (VDR) in dysplastic oral cells. VDR is widely expressed in most cell types, and its expression is progressively reduced during tumor progression in many cancer types [18,19,20,28,34]. Studies in melanoma cells have shown that 1,25-(OH)2D3 inhibits the Wnt/β-catenin pathway and cell growth in vitro and in vivo, and that VDR expression was significantly upregulated post-treatment [35]. By comparing VDR expression levels in normal, benign, and malignant tissues of skin, breast, ovarian and prostate, it was described a negative correlation between VDR expression and tumor malignancy [17,18]. Similarly, in colorectal cancer, low VDR expression is predominantly observed in patients with advanced cancer stages (III and IV), and interestingly, overexpression of VDR reduced β-catenin and Cyclin D1 levels, suggesting that the Wnt/β-catenin pathway is active during colorectal cancer because of reduced VDR [36]. In fact, tumor growth of the colon adenocarcinoma cell line SW480 in mice is impaired when cells overexpress VDR, indicating that VDR acts as a tumor suppressor in colorectal cancer [36]. Nuclear VDR expression has also been associated with better overall survival in lung and urothelial bladder cancer patients [20,23]. In our study, both oral dysplasia cells and oral explants from OPMD patients with histological diagnosis of dysplasia showed decreased nuclear expression of VDR. Interestingly, following treatment with 1,25-(OH)2D3, nuclear expression of VDR increased significantly. These observations suggest that VDR expression may be a valuable biomarker for the early diagnosis of OSCC. To complement these results, the effects of 1,25-(OH)2D3 on cell migration and viability were assessed. Here, we show for the first time that treatment of dysplastic oral cells with 1,25(OH)2D3 significantly decreased both migration and viability. In kidney cancer cells, it was shown that 1,25-(OH)2D3 inhibits migration and invasion by suppressing β-catenin [15]. Consistently, in our model, we showed that lithium-dependent activation of β-catenin prevents the reduced migration observed after treatment with 1,25(OH)2D3. Interestingly, the patients in our study displayed low serum vitamin D levels, which might be associated with the progression of their proliferative verrucous leukoplakias. Although the classical role of vitamin D is to regulate the metabolism of calcium and phosphate, extensive research has suggested that low sunlight exposure and vitamin D deficiency are also associated with an increased risk of cancer [37]. An inverse correlation between serum 25(OH)D levels and the high risk of developing colon, breast, prostate, and gastric cancer, among others, has been proposed. Epidemiologic studies suggest that low vitamin D levels are associated with an increased risk of OSCC [24,38,39]. However, the mechanism by which 1,25-(OH)2D3 inhibits migration is unknown.

The strengths of this study include the use of human in vitro and ex vivo models of oral dysplasia from similar anatomical locations (tongue), as well as the use of 1,25-(OH)2D3 0.1 µM, equivalent to 41.7 ng/mL, within the physiological range for calcitriol in serum [40]. A limitation of this study, however, is that it remains unknown whether the use of 1,25-(OH)2D3 could reduce other β-catenin-dependent malignant traits, such as cell invasion. Future studies will address these possibilities, as well as the role of 1,25-(OH)2D3 during oral carcinogenesis in vivo and in novel in vitro preclinical models. Indeed, DOK spheroids treated with 4-nitroquinoline 1-oxide (4NQO)—a carcinogen that mimics the effects of cigarette smoking—represent an in vitro 3D model of oral carcinogenesis [41]. This model is suitable to evaluate the area and length of the spheroid invasion after challenge with a drug, to assess the chemotherapeutic response in vitro [41]. Thus, it would be interesting to observe whether reduced spheroids invasion occurs after treatment with 1,25-(OH)2D3. Finally, given that 1,25-(OH)2D3 has synergistic effects on cytotoxicity when combined with other cancer treatments, i.e., chemotherapy, the results of this study open novel therapeutic approaches to prevent the progression from oral dysplasia to oral cancer.

## 5. Conclusions

In summary, this study shows that 1,25-(OH)2D3 treatment decreases migration and viability in oral dysplasia cells. This was accompanied by decreased Wnt/β-catenin activation. The results of our study suggest the potential of using 1,25-(OH)2D3 as a new therapeutic approach to prevent the progression of OPMDs with histological diagnosis of oral dysplasia towards OSCC by decreasing cell proliferation, cell migration, and cell viability, while inhibiting the Wnt/β-catenin pathway.

## Data Availability

Data are contained within the article.
